# Modeling fitness changes in wild Atlantic salmon populations faced by spawning intrusion of domesticated escapees

**DOI:** 10.1111/eva.12615

**Published:** 2018-03-23

**Authors:** Marco Castellani, Mikko Heino, John Gilbey, Hitoshi Araki, Terje Svåsand, Kevin A. Glover

**Affiliations:** ^1^ Department of Engineering University of Birmingham Birmingham UK; ^2^ Department of Biological Sciences University of Bergen Bergen Norway; ^3^ Institute of Marine Research Bergen Norway; ^4^ International Institute for Applied Systems Analysis (IIASA) Laxenburg Austria; ^5^ Freshwater Fisheries Laboratory Marine Scotland Pitlochry UK; ^6^ Research Faculty of Agriculture Hokkaido University Sapporo Japan

**Keywords:** admixture, aquaculture, farmed escapees, gene‐flow, genetic interactions

## Abstract

Genetic interaction between domesticated escapees and wild conspecifics represents a persistent challenge to an environmentally sustainable Atlantic salmon aquaculture industry. We used a recently developed eco‐genetic model (IBSEM) to investigate potential changes in a wild salmon population subject to spawning intrusion from domesticated escapees. At low intrusion levels (5%–10% escapees), phenotypic and demographic characteristics of the recipient wild population only displayed weak changes over 50 years and only at high intrusion levels (30%–50% escapees) were clear changes visible in this period. Our modeling also revealed that genetic changes in phenotypic and demographic characteristics were greater in situations where strayers originating from a neighboring wild population were domestication‐admixed and changed in parallel with the focal wild population, as opposed to nonadmixed. While recovery in the phenotypic and demographic characteristics was observed in many instances after domesticated salmon intrusion was halted, in the most extreme intrusion scenario, the population went extinct. Based upon results from these simulations, together with existing knowledge, we suggest that a combination of reduced spawning success of domesticated escapees, natural selection purging maladapted phenotypes/genotypes from the wild population, and phenotypic plasticity, buffer the rate and magnitude of change in phenotypic and demographic characteristics of wild populations subject to spawning intrusion of domesticated escapees. The results of our simulations also suggest that under specific conditions, natural straying among wild populations may buffer genetic changes in phenotypic and demographic characteristics resulting from introgression of domesticated escapees and that variation in straying in time and space may contribute to observed differences in domestication‐driven introgression among native populations.

## 1 INTRODUCTION

Atlantic salmon (*Salmo salar* L.) aquaculture was initiated in the early 1970s and has grown into an internationally significant industry with a worldwide production exceeding 2.3 million tons in 2016 (FAO 2016). Thus, Atlantic salmon aquaculture contributes significantly to the global blue revolution. Nevertheless, the industry′s exponential growth in the past decades has not been without environmental challenges. Of these, farmed escapees and subsequent genetic interactions with wild conspecifics (Glover et al., 2017) represent one of the most persistent and significant issues (Forseth et al., 2017; Taranger et al., 2015).

Each year, thousands or hundreds of thousands of domesticated salmon escape from farms into the wild (Skilbrei, Heino, & Svåsand, 2015). While most of these escapees are never seen again, presumably falling foul to predation, starvation or similar, some enter rivers and find their way onto the spawning grounds of native populations. A significant proportion of the spawning population in some rivers in some years is made up of domesticated escapees (Fiske, Lund, & Hansen, 2006; Morris et al., 2008; Saegrov, Hindar, Kalas, & Lura, 1997), and as a result of introgression, molecular genetic changes have been documented in native salmon populations in Ireland (Clifford, McGinnity, & Ferguson, 1998a; Clifford, McGinnity, & Ferguson, 1998b; Crozier, 1993, 2000), Norway (Glover et al., 2012, 2013; Karlsson, Diserud, Fiske, & Hindar, 2016; Skaala, Wennevik, & Glover, 2006), Scotland (Verspoor, Knox, & Marshall, 2016), and Canada (Bourret, O'Reilly, Carr, Berg, & Bernatchez, 2011). Atlantic salmon have been subject to targeted breeding programs from the very beginning of the industry, including selection for a wide range of economically important traits such as increased growth rates, delayed onset of maturation, and disease resistance (Gjedrem, 2000, 2010). Consequently, Atlantic salmon is regarded as one of the most domesticated aquaculture species globally (Teletchea & Fontaine, 2014) and displays genetic differences to wild salmon in a range of traits (Glover et al., 2017). Therefore, the widespread introgression of domesticated escapees in native populations is a cause of concern for the evolutionary trajectory and long‐term viability of wild populations (McGinnity et al., 2003).

Field experiments have demonstrated that the offspring of domesticated salmon, and hybrids between domesticated and wild salmon, display reduced survival in the wild when compared to the offspring of pure wild salmon (Fleming et al., 2000; McGinnity et al., 1997, 2003; Skaala et al., 2012). Furthermore, differences in phenotypic characteristics such as size at maturation have been reported between admixed and nonadmixed salmon in Norwegian populations where introgression of domesticated fish has occurred (Bolstad et al., 2017). Nevertheless, in most regions outside Norway where domesticated and wild salmon also overlap, there are almost no estimates of gene‐flow, and the resulting biological consequences have not been investigated (Glover et al., 2017). Therefore, the use of models that permit investigation and quantification of potential impacts on native populations, and at the same time open the possibility to examine various management and introgression scenarios, can help shed light on the potential outcomes of unilateral gene flow from domesticated escapees to native populations.

A range of models and modeling scenarios have been published in the past decade or so for investigating genetic changes in salmon populations following gene‐flow from maladapted domesticated salmon, or similar evolutionary scenarios (Baskett, Burgess, & Waples, 2013; Baskett & Waples, 2013; Besnier, Glover, & Skaala, 2011; Castellani et al., 2015; Hindar, Fleming, McGinnity, & Diserud, 2006; Huisman & Tufto, 2012; Piou & Prevost, 2012; Tufto, 2017). However, no study has thus far modeled potential phenotypic changes in life‐history traits (e.g., size at age and age of maturation) in wild populations that are subject to spawning intrusion from domesticated escapees. IBSEM, an Individual‐Based Salmon Eco‐genetic Model, was recently developed to investigate genetic changes in wild populations following intrusion of maladapted conspecifics (such as domesticated escapees) and includes extensive incorporation of the genetic parameters linked to the key fitness traits reported to diverge between domesticated and wild conspecifics (Glover et al., 2017). The model includes, among other key attributes, a quantitative genetic component and estimates phenotypic and demographic changes that are of relevance when considering gene‐flow from domesticated to wild populations (Castellani et al., 2015).

Presently, Norway is the only country where estimates of domesticated salmon introgression exist for a large number of wild populations (Glover et al., 2013; Karlsson et al., 2016). Here, admixture levels ranging from ~0% to 50% have been reported in populations, sometimes varying considerably among rivers within regions. A range of factors may influence the level of domestic salmon introgression in native populations, such as the incidence of escapees (Glover et al., 2013; Heino, Svåsand, Wennevik, & Glover, 2015; Karlsson et al., 2016) and the density or abundance of the recipient wild population (Glover et al., 2012; Heino et al., 2015). Other biological and physical factors, for example, a difficult‐to‐pass waterfall in the lower stretches of the river, may hinder some escapees from getting onto the primary spawning grounds and thereafter interbreeding. Variations in these and other unidentified factors contribute to the observed interpopulation differences in introgression levels. Another possibility is that, given that not all wild salmon home back to their native rivers (Stabell, 1984), strayers from relatively unaffected wild populations could buffer genetic changes in close‐by populations that are subject to spawning intrusion of domesticated escapees.

The present study had three objectives. First, to gain a better understanding of the magnitude and timescale of changes in a range of phenotypic traits (survival and life‐history traits such as size at age and age of maturity), underlying genotypes, and resultant demographic trajectories (i.e., numbers of fish) in wild salmon populations faced with different levels of spawning intrusion from domesticated escapees and continual natural selection. Second, to investigate the potential for recovery in native populations following introgression of domesticated escapees. Third, to quantify the degree to which phenotypic and demographic changes in wild populations due to the introgression of domesticated escapees can be influenced by straying among wild populations and the genetic admixture characteristics of strayers.

## 2 METHODS

### 2.1 Overall study design

The study was divided into two parts. First, we conducted a sensitivity analysis to investigate how straying (and thereafter gene‐flow) from a neighboring wild population may potentially buffer domestication‐driven genetic changes in a focal wild population subject to spawning intrusion of farmed escapees. We used a factorial design with the following three fully crossed binary factors (in total 8 combinations, corresponding to the scenarios 1–8 in Table 1): (A) the number of domesticated escapees was either held stable (domesticated intruders = Fixed *N* equating to 50% intrusion in the first year and steadily increasing as the wild population declined), or varied in tandem with the focal wild population (domesticated intruders = Fixed 50%), (B) the number of strayers from a neighboring wild population was either held at a “Fixed *N*” or alternatively “Fixed % of the focal population”—thus declining if the focal population declined, and (C) the strayers from a neighboring wild population were either “not admixed” or “domestication‐admixed at the same degree as the focal population.”

**Table 1:** Factorial design of intrusion scenarios tested for the sensitivity analyses

(A) Domesticated salmon intrusion level in the focal wild population	(B) Number of wild strayers entering the focal wild population	(C) Allele frequency of the wild strayers entering the focal wild population	Scenario
50% of wild salmon returners	5% of wild salmon returners	Same as focal population	1
		Fixed (0.8)	2
	Fixed at *N* = 25	Same as focal population	3
		Fixed (0.8)	4
Fixed at *N* = 250	5% of wild salmon returners	Same as focal population	5
		Fixed (0.8)	6
	Fixed at *N* = 25	Same as focal population	7
		Fixed (0.8)	8

Second, we conducted an in‐depth examination of the two most contrasting scenarios emerging from the sensitivity analysis (scenario 5—neighbor wild population is both domestication‐admixed and declines in parallel with the focal wild population, and scenario 8—neighbor wild population is not admixed and does not change in parallel with the focal wild population). Both these scenarios were tested under three levels of domesticated salmon intrusion (set at a Fixed *N* for domesticated intruders that equates to 5%, 10%, and 30% intrusion in the first year of the model before any potential changes in the recipient wild population) and included a detailed examination of the outputs of IBSEM. These outputs included density of eggs deposited in the river, juvenile growth/size and density, smolt growth/size, age and density, number of returning adults, and their growth/size and age of maturation.

### 2.2 Key attributes of IBSEM

IBSEM has been extensively described (Castellani et al., 2015). However, some of the key attributes relevant for understanding outputs of the model and the underlying basis of the computations are also described here for clarity. The model splits the Atlantic salmon life cycle into the “embryo” (which includes egg to early parr in freshwater), “juvenile” (which includes parr to smolts in freshwater), and “adult” stages (which includes fish in the marine environment returning to their natal river as sexually mature spawners). It uses a set of fitness differentials sourced from published empirical data to describe the difference between domesticated and wild salmon during these various life‐history stages. Growth and survival during each stage are influenced by individual genotypes as well as being size and density dependent. The model also distinguishes between the sexes. Female fertility is weight‐dependent. Reproductive success of adult males increases with their length, but males can also reproduce as sexually mature parr during the juvenile stage. It is important to note that the spawning (reproductive) success of the domesticated escapees, relative to wild spawners, has been set to 30% and 5% for females and males, respectively. This is based upon empirical knowledge from spawning experiments (Fleming, Jonsson, Gross, & Lamberg, 1996; Fleming et al., 2000) and from an extensive summary of spawning success of domesticated and ranched salmon in the wild (Hindar et al., 2006). The consequence of the differential spawning success between domesticated escapees and wild salmon means that for any simulated intrusion level, domesticated gene‐flow will be lower. That is, given equal sex ratios, 10% and 50% intrusion levels equate to 1.75% and 8.75% genetic contribution per year from the domesticated escapees in our model. This may of course vary in time and space; however, in the model it has been held stable.

**Figure 1:**
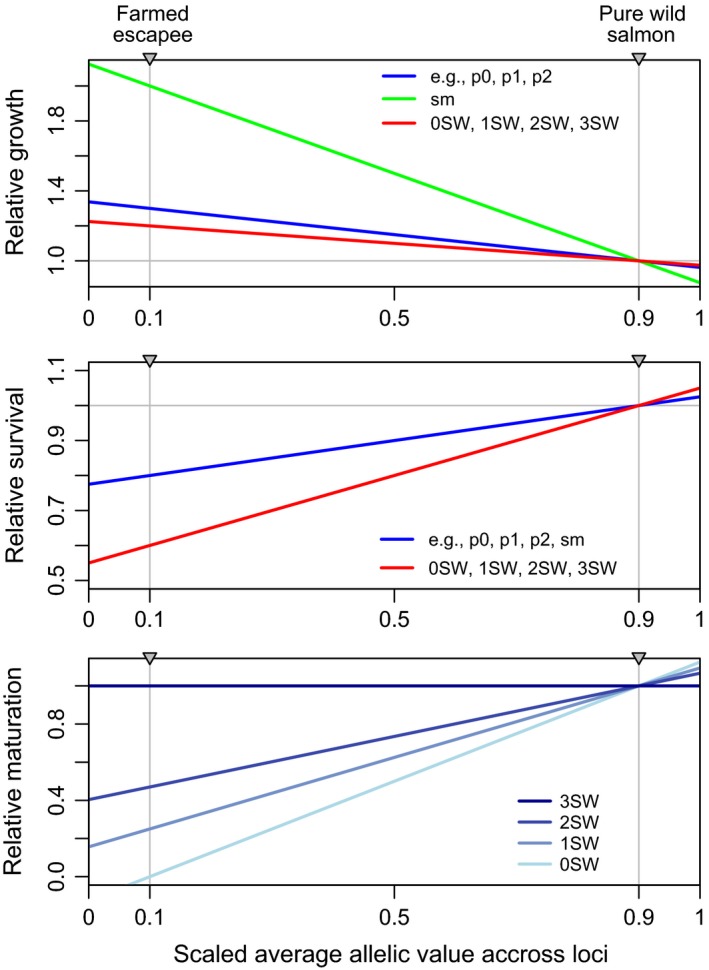
Genetic basis of the trait determination mechanism as implemented in the model IBSEM

The underlying genetic architecture of the model is determined by three independent sets of genes (influencing embryo, juvenile, and marine phase traits, respectively). Each of these sets of genes consists of 21 diploid and unlinked biallelic loci (alleles = 1 or 0, thus genotype combinations for each locus = 11, 10 or 00), with the ability for gamete recombination and random inheritance during sexual reproduction. Therefore, the random evolutionary force of genetic drift may play a role, especially when *N* is low. Loci 1–20 display an exponentially declining degree of influence over the phenotype from 15% for the 1st locus to 1% for the 20th locus. The 21st locus has no phenotypic impact and is selectively neutral (Fig. A in Castellani et al., 2015). The scaled average allelic value across loci of an individual (*S* ^ϕ^) is the sum of “1” alleles in a certain gene, weighted by loci‐specific degree of influence, and scaled such that the minimum (*S* ^ϕ^ = 0) and the maximum (*S* ^ϕ^ = 1) are obtained for, respectively, “00” and “11” homozygotes for all 20 loci of that gene. An individual could be anywhere on the scale between these two extremal values, depending on their genetic composition across these loci for each of the three independent sets of genes. The resultant genetic value, hereon referred to as the sum of the genetic effects, determines an individual's phenotypic value in terms of growth and survival for the three life stages in the model (embryo, juvenile, and adult). The genotype–phenotype maps are linear (Figure 1). For survival, this relationship is positive, that is, large genotypic values (wild alleles) are associated with high survival. For growth and maturation probability, the relationship is the opposite: wild alleles confer lower growth and earlier maturation. The specific equations and parameters estimates are given in Appendix 2 in Castellani et al. (2015).

The phenotypic differences between domesticated and wild salmon are expressed through their genotypes. Specifically, wild salmon in their native river were initiated with the allele frequency equal to 0.9 in all non‐neutral loci, whereas the farmed salmon were initiated with frequency 0.1 in all non‐neutral loci. The genotype–phenotype maps were tuned such that the average phenotype in wild salmon resulted in a population with demographic and life‐history characteristics similar to Atlantic salmon from the river Os in Norway (Castellani et al., 2015). Straying wild salmon (i.e., from a neighboring wild population) were created with the allele frequency equal to 0.8 in all non‐neutral loci. This gives slightly reduced fitness to wild strayers compared to wild salmon in their home river, as expected when individuals are locally adapted (Appendix 2 in Castellani et al., 2015).

Intrusion from domesticated spawners was simulated for 200 years, after which the focal wild population was permitted to recover for a further 200 years in their absence. For each scenario, ten independent runs were conducted and the results averaged. The simulations conducted are described in detail below and illustrated in Table 1.

### 2.3 The factorial design of intrusion scenarios 1–8

The primary objective of scenarios 1–8 was to simultaneously investigate the influence of the following factors and their potential interaction in a factorial experiment design: A. Fixed *N* vs. Fixed % domesticated intruders, B. Fixed *N* vs. Fixed % strayers from a neighbor wild population, C. Admixed vs. nonadmixed strayers from a neighbor wild population (Table 1). The rationale behind these scenarios, and how they were included in the experimental design, is described in detail below.

The first variable in the factorial setup was designed to investigate the influence that the number of intruding domesticated escapees had on the evolutionary trajectory of the focal wild population when held at a constant level (i.e., Fixed *N*) as opposed to varying in tandem with the focal wild population (i.e., Fixed %). In order to achieve this, intrusion of escapees was set at either 50% of the number of adults returning to the focal population each year (Fixed % scenarios 1–4), or set to a constant value of 250 for each year regardless of *N*‐adults returning to the focal population which corresponds to 50% in the first year of intrusion (Fixed *N* scenarios 5–8) (Table 1). While this reflects a very high level of domesticated salmon intrusion, some Norwegian rivers in some years have experienced this (Anon 2017; Fiske, Lund, Østborg, & Fløystad, 2001; Fiske et al., 2006), and based on earlier simulations (Castellani et al., 2015), this level of intrusion provides a response in the focal wild population thus making the potential influence of other parameters under investigation clearer on the simulated evolutionary timeline.

The second variable in the factorial setup was designed to investigate the influence that the number of fish straying from a neighbor wild population had on the evolutionary trajectory of the focal wild population when held at a constant level (i.e., Fixed *N*) as opposed to varying in tandem with the focal wild population (i.e., Fixed %). In order to achieve this, strayers were set at 5% of the *N*‐adults returning to the focal population (scenarios 1, 2, 5, 6), or fixed to 25, corresponding to 5% of the *N*‐adults returning to the focal population in the first year of intrusion (scenarios 3, 4, 7, 8). This level of straying is within the variation displayed between Atlantic salmon populations (Jonsson & Jonsson, 2017; Jonsson, Jonsson, & Hansen, 2003; Pedersen, Rasmussen, Nielsen, Karlsson, & Nyberg, 2007; Skilbrei & Holm, 1998; Stabell, 1984) and was designed to reflect situations where the neighbor population remained demographically stable over time and thus provided a constant number of strayers to the focal wild population, or alternatively, varied in tandem with the focal wild population, reflecting a situation where it is ecologically connected to the focal wild population.

The third variable in the factorial setup was designed to investigate the influence that the genetic composition of the straying fish from a neighbor wild population had on the evolutionary trajectory of the focal wild population when they are admixed at the same level as the adults returning to the focal wild population each year (scenarios 1, 3, 5, 7), or alternatively, not admixed with domesticated escapees and thus set to a fixed value of 0.8 (scenarios 2, 4, 6, 8). Both of these sets of scenarios, that is, admixed vs. nonadmixed strayers, are realistic given that in the wild, populations displaying both different and similar admixture levels can be observed in the same region (Glover et al., 2013; Karlsson et al., 2016).

For the full‐factorial design, results were expressed as the sum of the genetic effects in the model for the embryo, juvenile, and adult stages of the life cycle, and the demographic response of the adult spawning population, that is, the number of adults returning from the sea to spawn.

### 2.4 In‐depth analysis of intrusion scenarios 5 and 8

Following the initial sensitivity analyses as described above, two contrasting scenarios were chosen for an in‐depth analysis of the potential genetic changes in a wild population subject to spawning intrusion from domesticated escapees. These were scenario 5 whereby the neighbor population providing the strayers is ecologically linked and admixed at the same degree as the focal wild population itself (thus straying at 5% and admixed at the same degree as the wild population), and scenario 8, whereby the neighbor population providing the strayers is not ecologically linked nor admixed at the same degree as the focal wild population itself (thus straying at a Fixed *N* of 25 per year and not admixed). These two scenarios reflect the contrasting situations where there is global introgression in a region and all rivers are simultaneously influenced by intrusion of domesticated escapees (hereon referred to as “global”), as opposed to where there is only introgression in the focal wild population and the neighboring rivers are not influenced by intrusion of domesticated escapees (hereon referred to as “local”). “Global” and “local” reflect the two extreme points and were thus chosen to illustrate the potential influence that straying elicits on changes in populations influenced by intrusion of domesticated escapees.

For scenarios 5 and 8, the following output parameters were investigated: density of eggs deposited in the river, juvenile size in May, parr density and size in October (split by age), smolt density and size in May prior to migration from the river (split by age), number of adult salmon returning to the river to spawn, and their size (split by age). Domesticated salmon intrusion levels were set to *N* = 25, 50, and 150 reflecting 5%, 10%, and 30% intrusion of escapees in the first year of the model before any changes in the adult spawning population occurred. Pilot analyses using different intrusion levels revealed scaled effects of increasing intrusion levels, and thus, only results from these three levels of intrusion are presented.

## 3 RESULTS

### 3.1 Sensitivity testing (intrusion scenarios 1–8)

**Figure 2:**
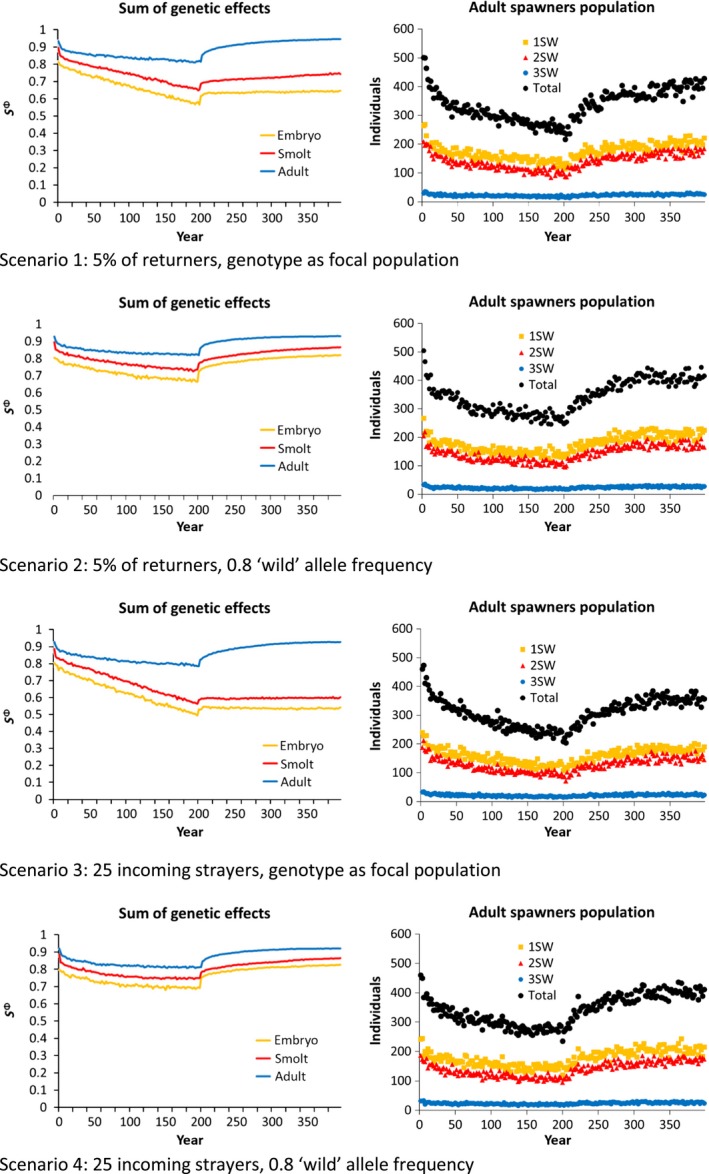
The Effect of different straying scenarios on the sums of genetic effects and demography of the focal wild population when the number of domesticated intruders is set to 50% of the focal population. Returning adults are broken down into their respective age groups

**Figure 3:**
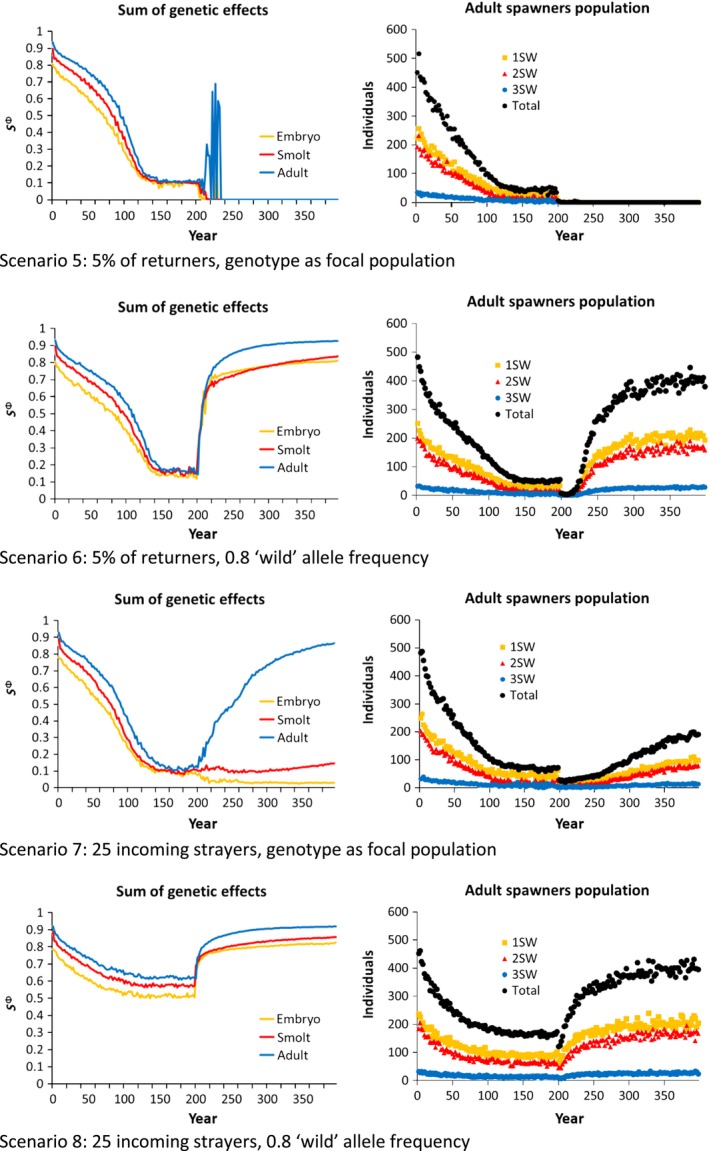
The effect of different straying scenarios on the sums of genetic effects and demography of the focal wild population when the number of domesticated intruders is set to *n* = 250 which equates to 50% of the focal population in the first year of simulation. Returning adults are broken down into their respective age groups

In all scenarios investigated, intrusion of domesticated escapees led to changes in the sums of genetic effects (i.e., the scaled average allelic values) in the direction of the domesticated genotype, reflecting introgression in the focal wild population (Figures 2, 3). These changes were accompanied by a decrease in the number of wild spawners returning to the focal population over time (Figures 2, 3). When intrusion of domesticated escapees was stopped at year 200, the genotypic values started to recover again, except for the most extreme scenario 5 leading to extinction of the focal population, and for next‐worse scenario 7, where the population persisted but significant genetic recovery only occurred for the adult traits.

The magnitude of change in the sums of genetic effects and the number of spawners returning to the focal population differed greatly between the intrusion scenarios. A clear split was observed between scenarios 1–4 vs. 5–8, simulating introgression of farmed escapees fixed as either 50% of the number of adults returning to the focal population (Figure 2), or fixed at *N* = 250 which is equal to 50% the number of wild spawners returning to the focal population in the first year of the simulation (Figure 3). The explanation for this difference is the self‐enforcing feedback loop coming from the negative effect that reduced‐fitness escapees have on population abundance in the focal river: as the abundance of the population gradually declines, the fixed number of escapees entering the river each year makes up an increasingly larger proportion of the remaining spawning population, leading to greater introgression and thereafter a further reduction in population abundance.

As genetic and phenotypic change was greatest in the Fixed *N* scenarios, making the potential influence of the other two factors in the factorial design, that is, straying rates from the neighboring wild population and their level of admixture, easier to detect, we here on concentrate on scenarios 5–8 (Figure 3). Based upon these simulations, a clear difference in both the sums of genetic effects and the rate in decline of the number of wild spawners were observed between scenario 5 where strayers from the neighboring wild population occurred at 5% of the focal wild population and were simultaneously domestication‐admixed at the same level of the wild population (global introgression), vs. scenario 8 where strayers from the neighboring wild population remained stable at 25 each year and were not admixed by domesticated escapees (local introgression). The difference between these scenarios was not distinct during the first ~60 years, where both resulted in a drop in the number of returning wild spawners to approximately 200. However, from ~60 years and onwards, the difference in the magnitude of effect between these scenarios was increasingly clear, resulting in a total collapse of the adult population in the global introgression scenario, while the local introgression scenario stabilized at 200 returning adults.

The large difference between these scenarios was caused by the difference in the degree of buffering provided by the neighboring wild population when it was ecologically disconnected from the focal population and not admixed (i.e., local introgression: the neighboring wild population provides a distinct buffering effect against domestication‐driven changes), compared to when it was ecologically connected to the focal wild population and equally admixed (i.e., global introgression: the neighboring wild population does not provide a distinct buffering effect against domestication‐driven changes).

### 3.2 In‐depth analysis of introgression (scenario 5‐global and 8‐local intrusion)

**Figure 4:**
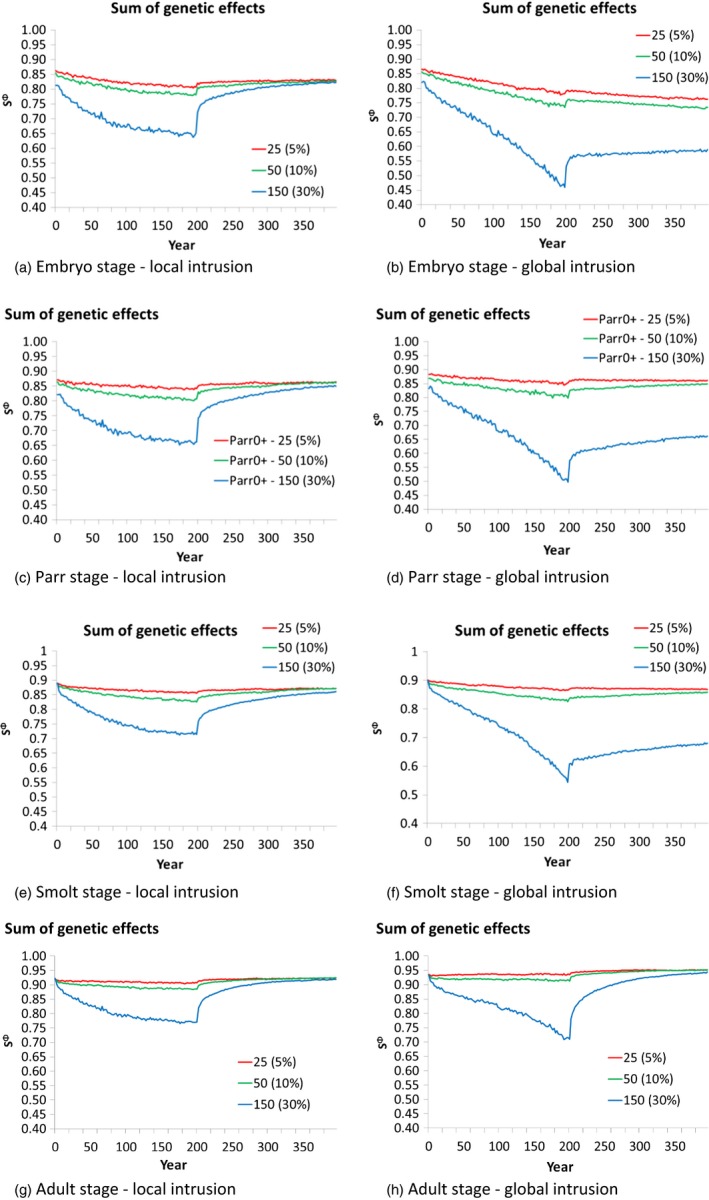
Sums of the genetic effects on the different life stages when the numbers of domesticated intruders was fixed to 5%, 10% and 30% of the numbers of adults in the focal population under the global and local intrusion scenarios (scenarios 5 and 8). For the parr stage, the plots show the results for the 0+ age group (the largest in population size). Similar trends were found for the 1+ and 2+ age groups

**Figure 5:**
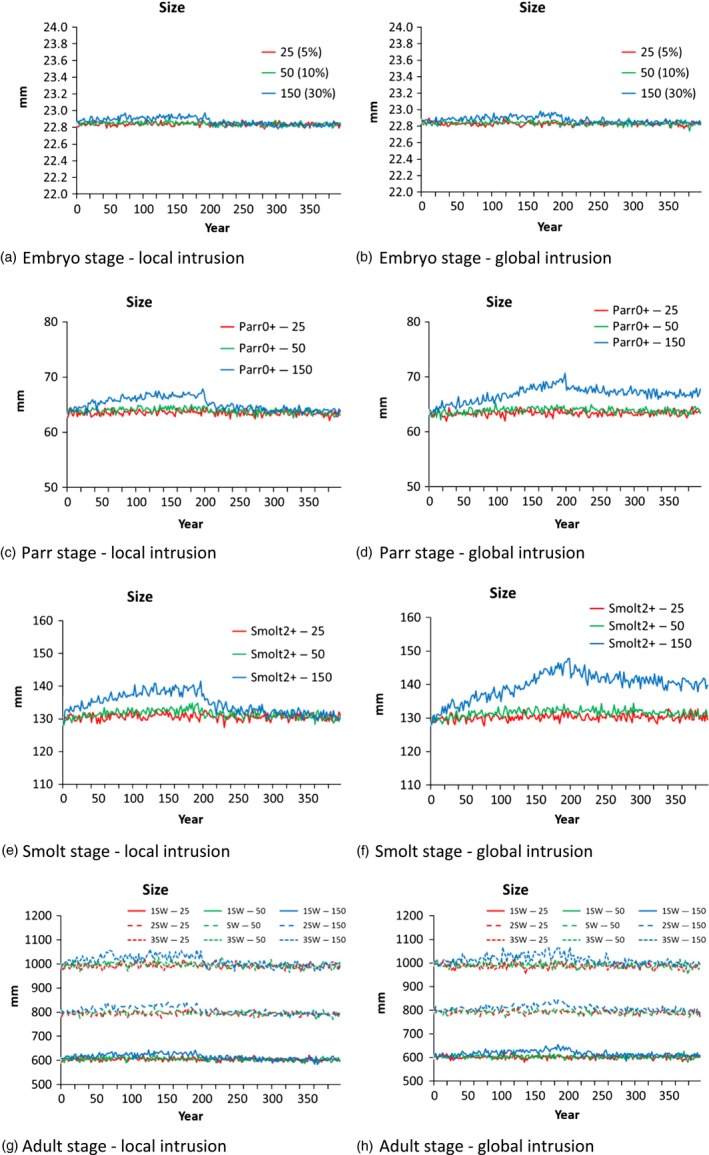
Size of salmon in the focal wild population at the different life stages when the number of domesticated intruders was fixed at 5%, 10%, and 30% of the focal wild population, for the global and local intrusion scenarios (scenarios 5 and 8). For the parr and smolt stages, the plots show the results for the 0+ and 2+ age groups, respectively (the largest in population size). Similar trends were found for the parr1+ and parr2+, and smolt1+ and smolt3+ age groups

**Figure 6:**
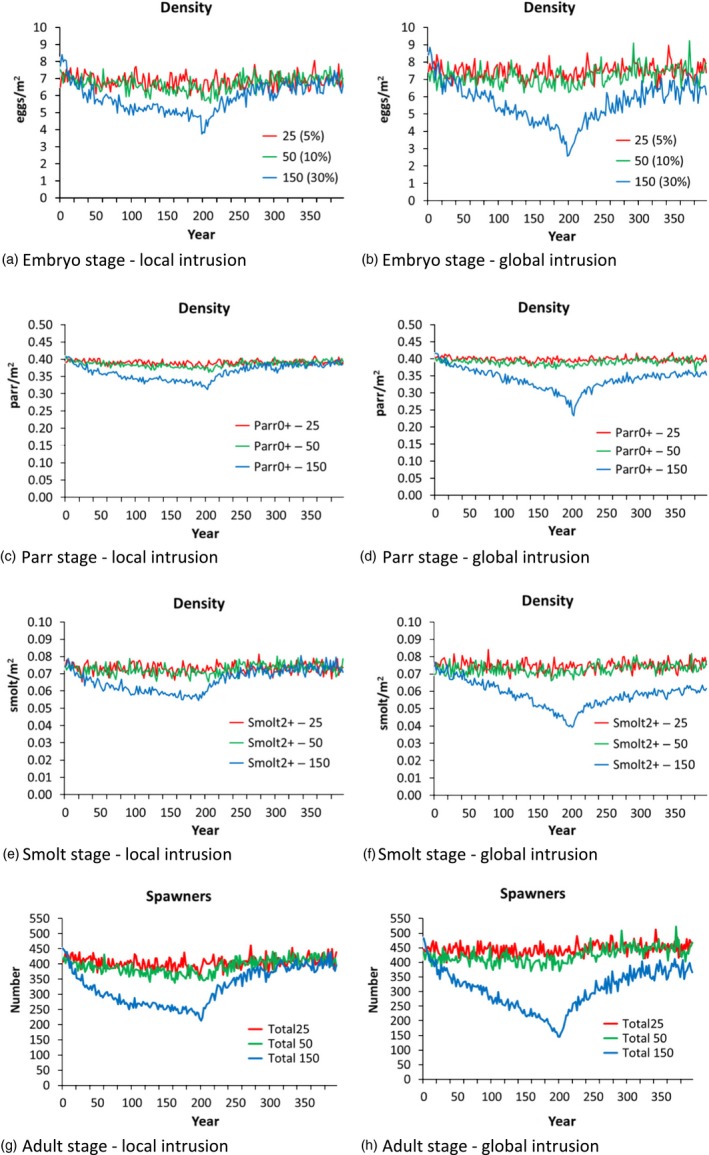
Density and number of salmon in the focal wild population at the different life stages, when the number of domesticated intruders was fixed at 5%, 10%, and 30% of the focal wild population under the global and local intrusion scenarios (scenarios 5 and 8). For the parr and smolt stages, the plots show the results for the 0+ and 2+ age groups, respectively (the largest in population size). Similar trends were found for the parr1+ and parr2+, and smolt1+ and smolt3+ age groups

Two contrasting scenarios emerging from the sensitivity analyses described above (global and local intrusion) were chosen for an in‐depth analysis of the phenotypic and demographic response of wild populations subject to spawning intrusion of domesticated escapees (Figure 4, 5, 6). In both scenarios tested, and as expected, higher frequencies of intrusion from domesticated escapees (5%, 10%, and 30%) led to a greater and more rapid response in the sums of the genetic effects (Figure 4), the degree of change in the phenotypic traits studied (Figure 5), and population demography (Figure 6). These changes were detected in both the local and global intrusion scenarios, but were greater in the global scenario due to the lack of buffering as described in the previous section. In most cases, spawning intrusion set at 5% and 10% domesticated individuals only led to small changes in the focal wild population, even over the 200‐year intrusion period, while the effect at 30% spawning intrusion was distinct for most of the traits, again, especially for the global intrusion scenario. Thus, for our study scenarios, natural selection was relatively effective in selectively removing low‐fitness alleles coming from the escapees when intrusion was low to moderate, but was gradually overwhelmed for the high level of intrusion.

Looking closer at the sums of the genetic effects (Figure 4), it is possible to see that the average genotype of the focal wild population changed in the direction of the domesticated genotype (i.e., decreasing from 0.9 toward 0.1) in all four stages of the life cycle where this was measured (embryo, parr, smolt, and adult). Turning attention to the phenotypic traits, most displayed a change over time (Figure 5). However, these changes were subtle for 5% and 10% intrusion of domesticated escapees. After 200 years, most of the changes in fish size at age were <10%, even for the strongest intrusion scenario run (30% intrusion). The greatest change was detected in the size of smolts exiting the river, especially for the older smolts (Figure 5). Changes in the size of adults in the focal wild population were weak for all sea ages (1+, 2+, 3+).

In part due to demographic stochasticity, the effects of the escapees on population demography were hardly discernible when intrusion was set at 5% and 10% (Figure 6). Concentrating on 30% spawning intrusion of domesticated escapees in the global scenario, the density and/or number of fish in the focal wild population showed a clear decrease with time in all stages measured (egg deposition, parr, smolts, adults returning to spawn), falling to less than half of the population's initial numbers over a 200‐year period.

## 4 DISCUSSION

This is the first study to model a set of phenotypic and demographic characteristics in an Atlantic salmon population subject to spawning intrusion by maladapted domesticated escapees. The results of our simulations indicate that at domesticated salmon spawning intrusion levels of 5% and 10% (~1%–2% gene‐flow per year), most of the phenotypic and demographic traits measured in the recipient wild population only displayed weak changes on a relatively short time scale (i.e., 50 years). Only when the frequency of domesticated escapees on the spawning grounds was increased to 30% or 50% (~5%–9% gene‐flow per year), were phenotypic and demographic changes clearly visible in the recipient wild population on this time scale. Based upon results from these simulations, together with existing knowledge, we suggest that a combination of reduced spawning success of domesticated escapees, natural selection purging maladapted phenotypes/genotypes from the wild population, and phenotypic plasticity buffer the rate and magnitude of change in phenotypic and demographic characteristics of wild populations subject to spawning intrusion of domesticated escapees. These suggestions are in line with empirical data demonstrating that domesticated escapees display reduced spawning success in comparison with wild salmon (Fleming et al., 1996, 2000), the offspring of domesticated salmon display reduced survival in the wild when compared to wild fish (Fleming et al., 2000; McGinnity et al., 1997, 2003; Skaala et al., 2012) and that differences between domesticated and wild salmon in phenotypic traits, such as growth rate, are much less pronounced in the wild than in the hatchery (Besnier et al., 2015; Jonsson & Jonsson, 2017; Reed et al., 2015; Skaala et al., 2012).

Our simulations also revealed that there is a difference in the rate of domestication‐driven genetic change in a wild population subject to escapees, when strayers arising from a neighboring wild population are simultaneously influenced by domesticated escapees (i.e., domestication‐admixed and decline at the same trajectory as the focal population—reflecting a global introgression scenario), as opposed to uninfluenced (i.e., not admixed and remain stable over time—reflecting introgression only in the focal wild population). This result is intuitive and suggests that unaffected (i.e., nonadmixed) neighboring populations may provide a partial buffer against fitness changes in wild Atlantic salmon populations invaded by domesticated escapees. In turn, we suggest that variation in population connectivity in time and space, and regional differences in the degree to which introgression of domesticated salmon occurs globally or locally, may contribute to the overall picture of large interpopulation patterns of domestication‐driven introgression that has been reported among wild Atlantic salmon populations (Glover et al., 2012, 2013; Karlsson et al., 2016).

### 4.1 Can variation in straying (buffering) contribute to interpopulation patterns in domestication‐driven admixture?

Connectivity among anadromous salmonid populations varies in time and space and is modified by a wide range of biotic and abiotic factors (Dillane et al., 2007; Dionne, Caron, Dodson, & Bernatchez, 2008; Glover et al., 2012; Perrier, Guyomard, Bagliniere, & Evanno, 2011). Within regions displaying a mosaic of small and large populations, straying and the degree of genetic connectivity may be skewed, whereby large populations can act as net‐exporters of strayers to smaller neighboring wild populations (Hansen, Skaala, Jensen, Bekkevold, & Mensberg, 2007). Straying of wild fish between rivers (Stabell, 1984) also varies in time and space for Atlantic salmon (Jonsson & Jonsson, 2017; Jonsson et al., 2003; Pedersen et al., 2007; Skilbrei & Holm, 1998) and other salmonids (Bett, Hinch, Burnett, Donaldson, & Naman, 2017; Ford, Murdoch, & Hughes, 2015; King, Hillman, Elsmere, Stockley, & Stevens, 2016). Recent experimental work has also indicated that introgression of domesticated salmon may increase straying rates in impacted wild Atlantic salmon populations (Jonsson & Jonsson, 2017).

In Norway, which is the country that has by far the greatest level of documentation of genetic interactions between domesticated escapees and wild conspecifics (Glover et al., 2017), estimates of domesticated salmon introgression levels now exist for nearly 200 populations (Diserud, Hindar, Karlsson, Glover, & Skaala, 2017; Glover et al., 2013; Karlsson et al., 2016). For many of these, estimates of the incidence of domesticated escapees are available for many years (Fiske et al., 2006). Using these and other population‐specific data, several authors have noted that both the magnitude of temporal genetic change (Glover et al., 2012) and the estimated level of cumulative introgression from domesticated escapees (Glover et al., 2013; Heino et al., 2015; Karlsson et al., 2016) are associated with the observed incidence of domesticated escapees. Nevertheless, the analyses performed in those studies all revealed that other factors may account for up to ~50% of the variation in introgression levels observed among wild populations. While the density (Glover et al., 2012, 2013) and/or numerical size (Heino et al., 2015) of the native population has been suggested to be a factor influencing the relative success of domesticated escapees, presumably through variations in spawning and/or juvenile competition, other factors (including their interactions) must also play a role in shaping the interpopulation differences observed in introgression levels.

A range of population‐biological and river‐physical parameters can potentially contribute to the observed patterns among populations in domestication‐driven admixture rates as discussed above. However, results from the modeling conducted here indicate that in cases where wild strayers are equally domestication‐admixed as the focal wild population, the degree of buffering against genetic changes in the direction of domesticated genome is much less than when the strayers are not admixed. In the wild, this could translate into regional “hot‐spots” of introgression where escapees swamp most or all rivers in a region and thus reduce the potential buffering effect from straying between rivers. Analyses of introgression in Norway have revealed for example that many rivers in the Hardangerfjord, a region of intense aquaculture production and characterized by large numbers of escapees over a long period of time, are strongly admixed with farmed escapees (Diserud et al., 2017; Glover et al., 2013; Karlsson et al., 2016). The fact that many of the rivers in that region are strongly affected by escapees means that there is very little potential for buffering from straying and therefore could contribute to the large admixture levels observed in the rivers of this region.

Atlantic salmon populations may display adaptations to the freshwater environments they inhabit in a process known as local adaptation (Garcia de Leaniz et al., 2007; Taylor, 1991). Reciprocal transplantation experiments conducted in the wild with Atlantic salmon have revealed differences in survival supporting this theory (McGinnity et al., 2004). In IBSEM, the potential for local adaptation is accounted for by coding the incoming strayers from the neighboring wild population as having a genetic value equal to 0.8 which equates to a slightly lower fitness of the incoming strayers than wild fish (0.9) in the focal population. We acknowledge that this is simplistic and may not necessarily reflect the true variation in fitness differences between two wild populations, and how this may or may not vary in time and space.

### 4.2 How do the model′s outputs compare to empirical data?

Our modeling reported weak population‐average changes in most of the traits studied in the first 50 years of simulated intrusion of domesticated escapees, especially at yearly domesticated salmon intrusion levels of 5%–10% (Figures 5, 6). Only when intrusion levels were set to a relatively high level (30% or 50%), and/or the timescale of investigation was extended to 200 years, were major changes in the focal wild population clearly evident. A similar pattern was also revealed in the validation scenarios presented earlier when the model was established (Castellani et al., 2015).

At the present, there are very few empirical data from wild populations with which to compare the projected changes from the modeling here. Nevertheless, there are experimental data which may provide us with clues. Domesticated Atlantic salmon display a wide range of genetic differences to wild salmon, and of these, growth and size at age are the traits displaying the greatest difference detected thus far (Glover et al., 2017). However, while very large (several fold or more in many cases) differences in growth and size at age are detected among domesticated, hybrid and wild salmon under controlled hatchery conditions (Debes, Fraser, Yates, & Hutchings, 2014; Fleming, Agustsson, Finstad, Johnsson, & Bjornsson, 2002; Glover et al., 2009; Harvey, Glover, Taylor, Creer, & Carvalho, 2016; Solberg, Glover et al., 2013; Solberg, Zhang et al., 2013), in the wild, growth rates and size at age differences between domesticated and wild salmon are much less distinct and overlap (Besnier et al., 2015; Fleming et al., 2000; Jonsson & Jonsson, 2017; Reed et al., 2015; Skaala et al., 2012). For example, size differences between domesticated and wild salmon in the river Burrishoole egg planting experiments conducted in Ireland in the late 1990s and early 2000s were reported to range from 5% to 20% (Reed et al., 2015), and the smolt size at age differences between domesticated, hybrid, and wild salmon from the egg planting experiments conducted in the river Guddal in Norway in the middle 2000s was typically only 0%–10% (Skaala et al., 2012). These estimates overlap with the relatively weak population‐average changes reported by the model here (Figure 5) and collectively indicate that at low to modest introgression rates, large differences in growth or size at age are not necessarily to be expected in wild populations. In addition to the lower spawning success of the farmed escapees (Fleming et al., 1996, 2000) limiting gene‐flow, we suggest that this is likely to be influenced by the generally higher mortality of the offspring of domesticated fish and hybrids in the wild (Fleming et al., 2000; McGinnity et al., 2003; Skaala et al., 2012). That is, as domesticated and domestication‐admixed fish hatched in the wild display higher mortality than wild fish, they will contribute less to the population‐level phenotypic changes.

A recent study reported phenotypic differences between domestication‐admixed and nonadmixed Atlantic salmon in populations where introgression of domesticated escapees had occurred (Bolstad et al., 2017). These authors investigated both age and size upon age at return to freshwater by categorizing fish within populations into domestication‐admixed and wild components using diagnostic genetic markers and a statistical method for computing admixture (Karlsson, Diserud, Moen, & Hindar, 2014; Karlsson, Moen, Lien, Glover, & Hindar, 2011). They generally observed higher sizes at age for fish classified as domestication‐admixed as opposed to fish classified as nonadmixed (wild). However, differences between the domestication‐admixed and wild salmon (both magnitude and direction) were dependent both upon the age category of the fish, and also, the geographic region in which admixture occurred (i.e., northern Norway contra middle/southern Norway). Others have concluded that growth differences between domesticated, hybrid, and wild salmon in the marine phase are not necessarily very clear, with small differences in maximum size at age but significant differences in minimum size at age (Jonsson & Jonsson, 2017). While the response in adult size in the IBSEM outputs reported here is low in comparison with the observations between domestication‐admixed and nonadmixed salmon (Figure 5 vs. results by Bolstad et al., 2017), it is pertinent to point out that the model outputs here reflect the population average. We did not separate out domestication‐admixed and nonadmixed fish as was the case in the analysis by Bolstad and colleagues (Bolstad et al., 2017). It is therefore suggested that the response revealed here is not necessarily very different to the observations made by Bolstad and colleagues, although this requires further investigation.

One of the most distinctive changes reported by the present modeling work was the decline in the number of adult spawners returning to the focal population. This negative additive effect is consistent with the “extinction‐vortex” theory as was first suggested following the two‐generation study of domesticated, hybrid, and wild salmon in the Burrishoole system in Ireland (McGinnity et al., 2003). Indeed, one of our most extreme intrusion scenarios tested also led to extinction (scenario 5, Figure 3). Statistically examining demographic population changes in the large number of Norwegian populations that have been subject to varying degrees of admixture (Diserud et al., 2017; Glover et al., 2013; Karlsson et al., 2016) may provide further insights into this.

In our model, we coded the spawning success of domesticated escapees as 30% and 5% for females and males, respectively, in relation to wild spawners. This was based upon data from experimental studies (Fleming et al., 1996, 2000) and overlaps with the values chosen in a model designed to investigate interbreeding between domesticated escapees and wild conspecifics (Hindar et al., 2006). However, it is pertinent to point out that the relative spawning success of domesticated escapees is likely to vary in time and space as introgression levels in wild populations have been suggested to be partially density dependent (Glover et al., 2012, 2013; Heino et al., 2015), and because their relative success is likely to be dependent on their time in the wild prior to spawning (Fleming, Lamberg, & Jonsson, 1997).

One of the most significant gaps in current knowledge, with respect to the genetic differences between domesticated and wild salmon in the wild, is their relative survival in the marine environment (Glover et al., 2017). The marine survival differential between offspring of domesticated and wild salmon used in IBSEM is based upon knowledge from the Burrishoole experiments in Ireland that compared hatchery‐reared smolts of local Irish wild salmon and nonlocal Norwegian domesticated salmon (thus mixing domestication and phylogenetic differences) (McGinnity et al., 1997, 2003). Other studies using domesticated and wild salmon from the same phylogenetic region have not reported such large differences in marine survival, although this varies (Fleming et al., 2000; Jonsson & Jonsson, 2017). Our modeling work indicates that the largest change to be expected in a wild population following spawning intrusion of domesticated escapees is in the number of adult spawners retuning to the river. Given that this is strongly influenced by the relative survival between offspring of domesticated and wild salmon, our work points to the need for further empirical data on this stage of the life cycle.

### 4.3 Implications of these results for mitigation and management

The results of the modeling described here have important implications for potential management strategies to mitigate the negative effects of domesticated‐mediated changes in wild populations faced with introgression of farmed escapees. As discussed above, outputs of the model indicate that in low‐to‐moderate spawning intrusion cases, and on a relatively short evolutionary time scale (e.g., ~50 years), the resulting changes in native populations are not expected to be very large, potentially making them difficult to detect (Figures 5, 6). Nevertheless, in the face of constant introgression, genetic changes are cumulative, and over time, significant demographic and fitness‐linked genetic changes are expected. This has also been indicated by other models which have looked at potential changes in the genetic composition and/or fitness consequences of wild populations faced by domesticated salmon introgression (i.e., from wild to admixed) (Baskett et al., 2013; Hindar et al., 2006). Clearly, managers and stakeholders need to be aware of the fact that genetic changes may accumulate over time and that the lack of any distinct change in population averages in the short‐term does not necessarily indicate that potentially negative fitness consequences on the wild population have not occurred.

The ultimate approach to protect native populations from further genetic changes from interbreeding of farmed escaped salmon is a significant reduction in the number of domesticated escapees and/or sterilization of farmed fish (Glover et al., 2017). The results of our modeling indicate that in regions where this is not immediately attainable, channeling efforts to protect large and regionally significant rivers, which can thereafter act as “wild gene‐banks” through natural straying and buffering as described here, may be worth considering. For example, in the Hardangerfjord, one of the regions in Norway most affected by introgression of farmed escapees, the largest population inhabits the river Etne. This population has been demonstrated to be admixed with farmed escapees at ~20% (Glover et al., 2013; Karlsson et al., 2016). Recently, an upstream trapping system that permits removal of nearly all escapees trying to enter the river was installed (Madhun et al., 2017; Quintela et al., 2016), thus offering the native population protection from further admixture and the ability to recover in a region where there are still persistently high numbers of farmed escapees. In the future, natural strayers from this recovering population may buffer domestication‐driven introgression in close‐by rivers.

Each year, the Institute of Marine Research conducts an environmental risk assessment of Norwegian aquaculture (Taranger et al., 2015). This exercise also includes escapees and genetic interactions with wild conspecifics. In the risk assessment, 10% incidence of farmed escapees has been set as the level which once exceeded, equates to a “large probability for genetic changes” in the native population. Here, we have demonstrated the ability of the recently developed model IBSEM (Castellani et al., 2015) to investigate the fitness‐related consequences of introgression in native populations. Future risk‐assessment exercises could incorporate results from modeling with IBSEM in order to assist in setting threshold levels.

## DATA ARCHIVING STATEMENT

There are no raw data associated with this work.

## CONFLICT OF INTEREST

None declared.
